# Glycemic control affecting the autonomic modulation in type 2 diabetes

**DOI:** 10.1186/1758-5996-7-S1-A3

**Published:** 2015-11-11

**Authors:** Daniela Bassi, Vivian Maria Arakelian, Renata Gonçalves Mendes, Flávia Cristina Rossi Caruso, Luis Carlos Bonjorno, Ross Arena, Audrey Borghi e Silva

**Affiliations:** 1 Universidade Federal de São Carlos, São Carlos, Brazil

## Background

Diabetes Mellitus (DM) is a chronic disease with high morbidity and mortality and one of the most important risk factors for developing cardiovascular disease. DM is associated with cardiac autonomic dysfunction.

## Objective

To evaluate if the glycemic control affects cardiac autonomic modulation in individuals with type 2 diabetes.

## Materials and methods

We evaluated 49 patients (51.2±7.7 yrs.) with a confirmed diagnosis of diabetes. The subjects were randomized in two groups according to glycated hemoglobin-HbA1c: HbA1c < 7% and HbA1c >7%. The fasting plasma glucose and HbA1c were performed in a specialized laboratory and HR and iRR were recorded for 10 min in the supine position. Statistical analysis included Shapiro-Wilk Test, Mann Whitney Test and Spearman Correlation.

## Results

Diabetics with HbA1c>7 presented lower values of all HRV indices compared to diabetics with HbA1c<7 (mean iRR=844.25±117.64 vs 928.47±67.83 ms; STDRR=21.13±12.85 vs 34.92±19.51 ms; RR Tri=5.57±3.07 vs 9.02±5.12; TINN=107.82±65.72 vs 149±50.32, SD2=37.70±19.11 vs 61.94±25.06 ms, except for mean HR (72.45±9.55 vs 65.12±4.91 bpm) where was higher in HbA1c>7 group. HbA1c was negatively correlated with mean iRR (r=-0.28); STDRR(r=-0.33); RRTri (r=-0.35), SD2(r=-0.39) and positively with mean HR (r=0.28). Whereas fasting plasma glucose was negatively correlated with SD2 (r=-0.42); STDRR(r=-0.36), RRTri (r=-0.36) and TINN (r=0.33).

## Conclusion

These findings suggest attenuated cardiac autonomic response in diabetics type 2 with poor metabolic control.

